# Supplementary material for: Short-Term Growth Hormone Administration Mediates Hepatic Fatty Acid Uptake and De Novo Lipogenesis Gene Expression in Obese Rats
Source: Biomedicines. 2023 Mar 29;11(4):1050. doi: 10.3390/biomedicines11041050 (PMC10135739; doi:10.3390/biomedicines11041050)

**Title:** Short-Term Growth Hormone Administration Reduces Hepatic Fatty Acid Uptake and *De Novo* Lipogenesis in Obese Rats

**Authors:** Sutharinee Likitnukul, Sumpun Thammacharoen, Orada Sriwatananukulkit, Chanathip Duangtha, Ruedee Hemstapat, Chotchanit Sunrat, Supachoke Mangmool and Darawan Pinthong *

**Type of manuscript:** Original Research Article

## Supplementary materials

**Figure S1.** a) The relative percentage of body fat mass in the control group; * represents statistical significance between GH and NSS groups ($p < 0.05$). b) The relative percentage of body fat mass in the DIO group. c) The relative percentage of body fat mass in the DR group. Isca: interscapular adipose tissue, Ing: inguinal adipose tissue, Mesen: mesenteric adipose tissue, Retro: retroperitoneal and perirenal adipose tissue, Epi: epididymal adipose tissue.

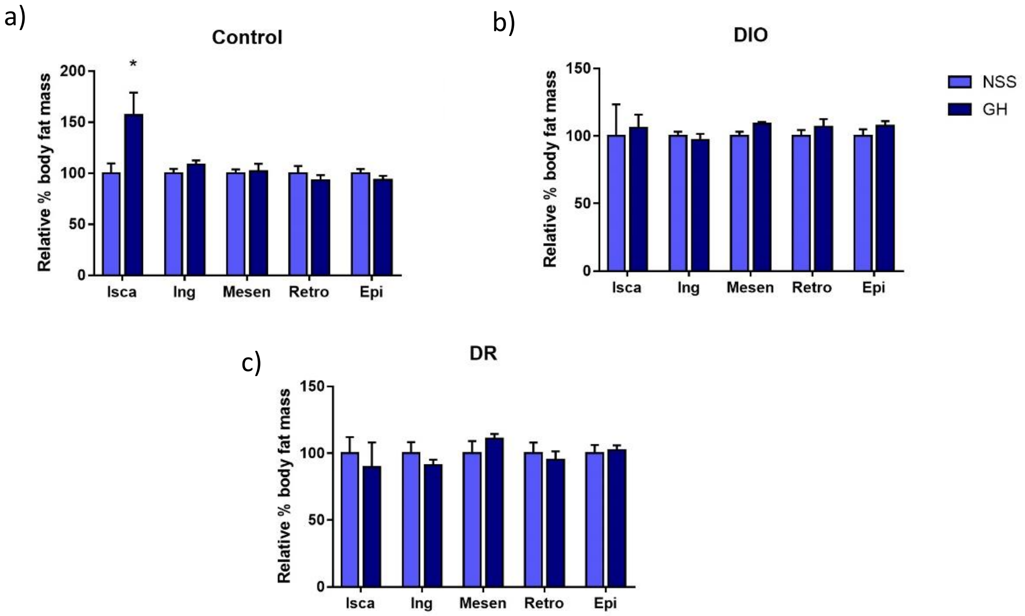

Supplement: Supplementary file 1 [file biomedicines-11-01050-s001.zip › biomedicines-2247959-supplementary.pdf]
